# Blood Pressure Associates with Standing Balance in Elderly Outpatients

**DOI:** 10.1371/journal.pone.0106808

**Published:** 2014-09-15

**Authors:** Jantsje H. Pasma, Astrid Y. Bijlsma, Janneke M. Klip, Marjon Stijntjes, Gerard Jan Blauw, Majon Muller, Carel G. M. Meskers, Andrea B. Maier

**Affiliations:** 1 Department of Rehabilitation Medicine, Leiden University Medical Center, Leiden, The Netherlands; 2 Department of Gerontology and Geriatrics, Leiden University Medical Center, Leiden, The Netherlands; 3 Department of Internal Medicine, Section of Gerontology and Geriatrics, VU University Medical Center, Amsterdam, The Netherlands; 4 Department of Geriatrics, Bronovo Hospital, The Hague, The Netherlands; 5 Department of Rehabilitation Medicine, VU University Medical Center, Amsterdam, The Netherlands; Medical University of Graz, Austria

## Abstract

**Objectives:**

Assessment of the association of blood pressure measurements in supine and standing position after a postural change, as a proxy for blood pressure regulation, with standing balance in a clinically relevant cohort of elderly, is of special interest as blood pressure may be important to identify patients at risk of having impaired standing balance in routine geriatric assessment.

**Materials and Methods:**

In a cross-sectional cohort study, 197 community-dwelling elderly referred to a geriatric outpatient clinic of a middle-sized teaching hospital were included. Blood pressure was measured intermittently (n = 197) and continuously (subsample, n = 58) before and after a controlled postural change from supine to standing position. The ability to maintain standing balance was assessed during ten seconds of side-by-side, semi-tandem and tandem stance, with both eyes open and eyes closed. Self-reported impaired standing balance and history of falls were recorded by questionnaires. Logistic regression analyses were used to examine the association between blood pressure and 1) the ability to maintain standing balance; 2) self-reported impaired standing balance; and 3) history of falls, adjusted for age and sex.

**Results:**

Blood pressure decrease after postural change, measured continuously, was associated with reduced ability to maintain standing balance in semi-tandem stance with eyes closed and with increased self-reported impaired standing balance and falls. Presence of orthostatic hypotension was associated with reduced ability to maintain standing balance in semi-tandem stance with eyes closed for both intermittent and continuous measurements and with increased self-reported impaired standing balance for continuous measurements.

**Conclusion:**

Continuous blood pressure measurements are of additional value to identify patients at risk of having impaired standing balance and may therefore be useful in routine geriatric care.

## Introduction

Five to 30 percent and 53 to 78 percent of elderly aged above 65 years suffer from orthostatic hypotension (OH) [1] and hypertension [2], respectively. Both OH and hypertension are signs of impaired blood pressure regulation [3], [4], which is associated with increased risk of cardiovascular events [5]–[7], falls [8]–[14], and mortality [15]–[18]. Another important risk factor of falls is impaired standing balance [13], [19], [20] resulting from the deterioration of underlying systems, i.e. the sensory systems (proprioception, vision and vestibular), muscles and neural control [21].

Few studies investigated the relation between blood pressure regulation and standing balance [22]–[25]. In healthy elderly aged above 65 years, hypertension was found to be unrelated to quality of standing balance measured by Center of Pressure (CoP) movement [24], but was related to the score on a dynamic pull test investigating postural stability [25]. Furthermore, in healthy elderly and patients with Parkinson's disease, OH was found to be associated with higher Center of Mass (CoM) movement during standing [22], [23].

In clinical practice, comparison of blood pressure measurements before and after a postural change from supine to standing position is used as a proxy for blood pressure regulation. In this study, we assessed the association of both intermittent and continuous blood pressure measurements before and after a postural change with three measures of standing balance: 1) the ability to maintain standing balance, 2) self-reported impaired standing balance and 3) history of falls, in community-dwelling elderly referred to a geriatric outpatient clinic. Results are relevant for design of routine geriatric assessment and therapeutic strategies.

## Materials and Methods

### Setting and study population

This cross-sectional study included 207 community-dwelling elderly who were referred to a geriatric outpatient clinic in a middle-sized teaching hospital (Bronovo Hospital, The Hague, Netherlands) for a comprehensive geriatric assessment (CGA) between March 2011 and January 2012. CGA was performed during a two hour visit including questionnaires and physical and cognitive measurements. All tests were performed by trained nurses or medical staff. The study was reviewed and approved by the institutional review board of the Leiden University Medical Center (Committee Medical Ethics (CME), Leiden, the Netherlands). The need for individual informed consent was waived, as this research was based on patient care. Ten elderly patients (4.8%) were excluded due to missing data on standing balance, leaving 197 patients for analyses. Continuous blood pressure measurements were added to the CGA in June 2012 and were subsequently available in 62 patients. Data of four patients were excluded because of technical problems, leaving 58 patients for analysis. Of two patients who visited the outpatient clinic twice, data were used from the second visit that included the continuous blood pressure measurements.

### Blood pressure measurements

Blood pressure was measured in supine position and during 3 minutes in standing position after postural change. Patients were in supine position for at least 5 minutes. An automatic lift chair (Vario 570, Fitform B.V., Best, The Netherlands) was used to provide automated support from a supine to a raised position. Subsequently patients were asked to stand up and stand unsupported for 3 minutes.

#### Intermittent blood pressure measurements

Systolic and diastolic blood pressure measurements were determined intermittently using an automated sphygmomanometer on the left arm (Welch Allyn, Skaneateles, USA). Blood pressure was measured after at least 5 minutes in supine position before postural change and after 1 and 3 minutes in standing position. Three blood pressure measures were determined: 1) supine blood pressure was defined as the blood pressure measured in supine position before postural change; 2) blood pressure decrease was calculated for two time points by subtracting the blood pressure taken at 1 or 3 minutes in standing position from the supine blood pressure; 3) OH_intermittent_ was defined as a decrease of at least 20 mmHg systolic blood pressure or 10 mmHg diastolic blood pressure at 1 or 3 minutes in standing position compared to supine blood pressure [26].

#### Continuous blood pressure measurements

At the same time, systolic and diastolic blood pressure measurements were determined continuously and non-invasively using a digital photoplethysmograph with a cuff placed on the right middle finger (Finometer PRO, Finapres Medical Systems BV, Amsterdam, The Netherlands) [27]. Data were analyzed using BeatScope 1.1 software (Finapres Medical systems BV, Amsterdam, The Netherlands) resulting in beat-to-beat blood pressure data. Beat-to-beat blood pressure data were exported to Matlab (The Mathworks, Natick, MA) and averaged over 5 seconds intervals [28]. Three blood pressure measures were determined: 1) supine blood pressure was defined as the mean blood pressure in supine position during the last 60 seconds before postural change; 2) blood pressure decrease was calculated for three consecutive time periods, i.e. 0 to 15 seconds, 15 to 60 seconds and 60 to 180 seconds after postural change by subtracting the lowest averaged blood pressure measured during the time period from the supine blood pressure; 3) OH_continuous_ was defined as a decrease of at least 20 mmHg systolic blood pressure or 10 mmHg diastolic blood pressure after 15 to 180 seconds in standing position compared to supine blood pressure. In addition, initial OH (iOH) was included in the definition of OH_continuous_ defined as a decrease of at least 40 mmHg systolic blood pressure or 20 mmHg diastolic blood pressure during the first 15 seconds compared to supine blood pressure [29], [30].

### Standing balance

The ability to maintain standing balance was assessed in three standing positions characterized by a progressive narrowing of the base of support performed both with eyes open and eyes closed. Patients, wearing non-slip socks, were instructed to maintain balance for 10 seconds in each standing condition. During side-by-side stance, patients were instructed to stand with the medial malleoli as close together as possible; during semi-tandem stance, with the medial side of the heel of one foot touching the big toe of the other foot; and during tandem stance, with both feet in line while the heel of one foot touched the toes of the other. Standing positions with eyes open were first assessed as part of the Short Physical Performance Battery (SPPB) [31]. Subsequently, all standing positions were repeated with eyes closed. Patients were allowed three trials if standing balance was lost prematurely. When the patients could not complete a standing position, consecutive positions were omitted. Six patients did not attempt the standing positions with eyes closed due to lack of time or lack of motivation, leaving 191 patients for analyses of standing balance positions with eyes closed. Impaired standing balance was self-reported by answering the question whether and how often the patient experienced problems with standing balance. A positive answer was registered when the answer option ‘regularly’ or ‘always’ was given. History of falls was self-reported by answering the question whether falls in the past 12 months were experienced.

### Characteristics of patients

Aforementioned items were part of a larger questionnaire obtaining information on marital status, living arrangements, smoking, alcohol use and use of walking aid. Body mass index was calculated by measuring body weight and height. Information on diseases and use of medication was extracted from medical charts. Multimorbidity was rated as the presence of two or more diseases including chronic obstructive pulmonary disease, heart failure, diabetes mellitus, hypertension, malignancy, myocardial infarction, Parkinson's disease, (osteo)arthritis, transient ischemic attack and stroke. The Hospital Anxiety Depression Scale (HADS) was used to detect depressive symptoms [32]; a score higher than 8 out of 21 points indicated depressive symptoms. Global cognitive functioning was assessed using the Mini Mental State Examination (MMSE) [33]. Handgrip strength was measured in standing position using a hand dynamometer (Jamar, Sammons Preston, Inc., Bolingbrook, IL, USA). The best performance of three trials alternately for each hand was used for analyses. Physical functioning was measured with a 10 meter walking test at usual pace in steady state, and with the SPPB. The SPPB comprises the ability to maintain balance in three standing positions with eyes open, a timed four meter walk and a timed sit-to-stand test.

### Statistical analyses

Continuous variables with Gaussian distribution are presented as mean and standard deviation; otherwise as number and percentage or median and interquartile range. The association between blood pressure measures and 1) the ability to maintain standing balance; 2) impaired standing balance; and 3) history of falls were analyzed using logistic regression models including adjustment for demographics, i.e. age and sex. P values less than 0.05 were considered statistically significant. Statistical analyses were performed using SPSS for Windows (SPSS Inc, Chicago, USA), version 20. For visualization purposes, tertiles of blood pressure decrease were calculated. Graphs were made with GraphPad Prism 5 (GraphPad Software, Inc., La Jolla, USA).

## Results

### Characteristics of patients

Characteristics of patients, including intermittent blood pressure measures, are presented in Table 1. Continuous blood pressure measures for the subgroup of patients are shown in Table S1. The mean age of all patients was 81.9 years. OH_intermittent_ was present in 29 out of 197 patients (15%). OH_continuous_ was present in 33 out of 58 patients (57%); in 19 patients (58%) also initial OH was present and in 5 patients (15%) only iOH was present. In 26 of 33 patients (79%) in which OH was present using continuous measurements, no OH was present using intermittent measurements.

**Table 1:** Characteristics of all elderly patients and of subgroup of elderly patients who underwent additional continuous blood pressure measurements.

	All (n = 197)	Subgroup (n = 58)
**Socio-demographics**		
Age, years	81.9 (7.1)	80.6 (7.0)
Men, n (%)	78 (39.6)	25 (43.1)
Widowed, n (%)	80 (41.5)	17 (29.8)
Independent living, n (%)	154 (79.4)	46 (79.3)
Current smoking, n (%)^a^	22 (16.2)	9 (15.5)
Excessive alcohol use, n (%)^e^	8 (4.1)	6 (10.3)
**Health characteristics**		
BMI, kg/m^2b^	25.8 (4.5)	26.4 (4.9)
Multimorbidity, n (%)^b, f^	95 (50.3)	26 (46.4)
Number of medication, median (IQR)^b^	5 (3–7)	5 (3–7)
HADS, depression > 8; n (%)^c^	28 (23.1)	10 (20.4)
MMSE, points; median (IQR)	27 (24–29)	28 (25–29)
**Physical functioning**		
Handgrip strength, kg	26.1 (8.2)	27.2 (7.9)
Gait speed, m/s	0.87 (0.29)	0.87 (0.29)
SPPB, points; median (IQR)	7 (5–10)	8 (6–10)
*Self-reported, n (%)*		
Fall incident previous 12 months	127 (64.5)	34 (58.6)
Impaired standing balance^g^	88 (45.1)	20 (35.1)
Use of walking aid	108 (55.1)	29 (50.0)
**Supine blood pressure** ^h, b^		
Systolic blood pressure, mmHg	142 (24)	141 (25)
Diastolic blood pressure, mmHg	74.6 (10.1)	74.4 (11.0)
**Blood pressure decrease after postural change**		
Orthostatic hypotension, n (%)^i^	29 (15.4)	7 (12.5)
*Systolic blood pressure decrease, mmHg^j, d^*		
1 minute	3.15 (15.94)	−0.62 (18.24)
3 minutes	−0.80 (15.55)	−4.37 (16.03)
*Diastolic blood pressure decrease, mmHg^j, d^*		
1 minute	−2.90 (7.18)	−4.53 (7.10)
3 minutes	−4.17 (8.27)	−5.76 (9.54)

All parameters are presented as mean with standard deviation unless indicated otherwise. Data available in ^a^ n = 136, ^b^ n = 190, ^c^ n = 121, ^d^ n = 181. ^e^ Defined as > 14 units per week for females and > 21 units per week for males. ^f^ Present in case of two or more diseases, including chronic obstructive pulmonary diseases, heart failure, diabetes mellitus, hypertension, malignancy, myocardial infarction, Parkinson's disease, (osteo)arthritis, transient ischemic attack and stroke. ^g^ Defined as regularly or always self-reported impaired standing balance. ^h^ Measured after at least 5 minutes in supine position. ^i^ Orthostatic hypotension defined as decrease in systolic blood pressure of ≥ 20 mmHg or decrease in diastolic blood pressure of ≥ 10 mmHg at 1 or at 3 minutes after postural change, intermittently measured. ^j^ Supine blood pressure minus blood pressure at 1 or 3 minutes after postural change. IQR: inter quartile range. BMI: Body Mass Index. HADS: Hospital Anxiety and Depression Scale. MMSE: Mini Mental State Examination. SPPB: Short Physical Performance Battery.

### Standing balance

**Figure 1:**
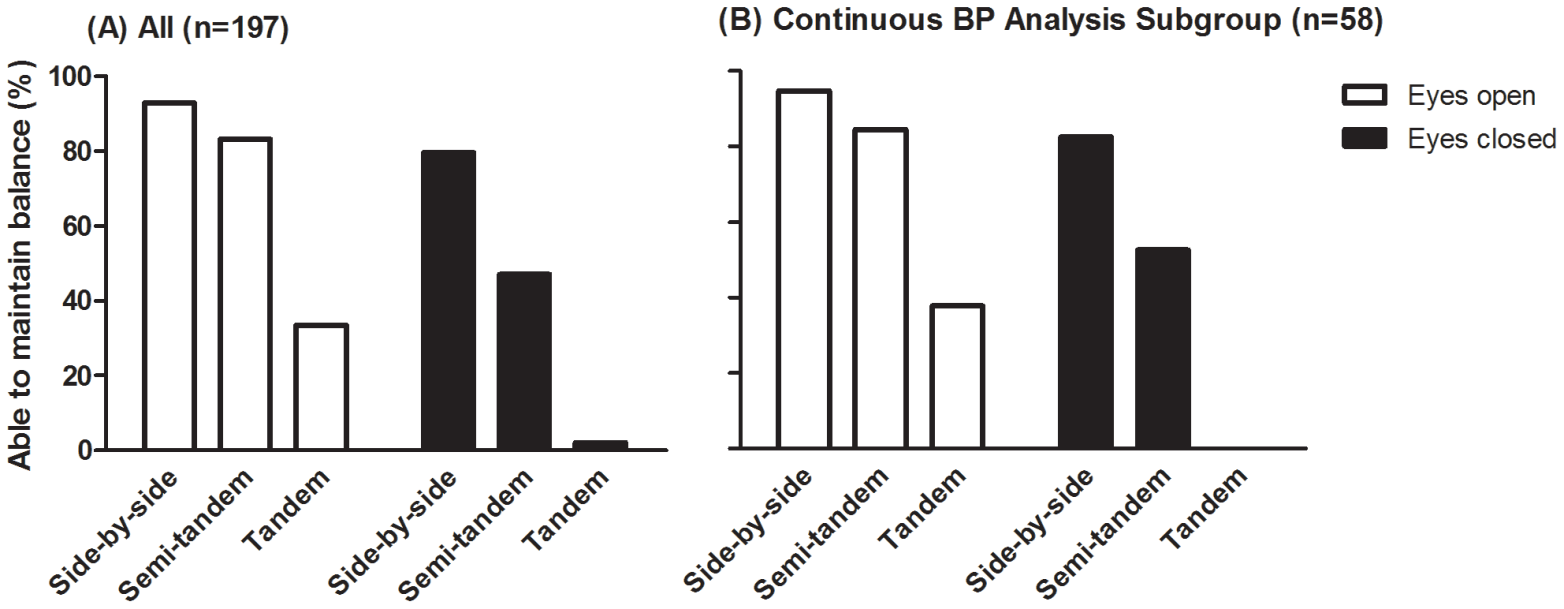
Ability to maintain balance in several standing positions with eyes open and eyes closed. A) all elderly patients (n = 197) and for B) subgroup who underwent additional continuous blood pressure measurements (n = 58).

Ability to maintain standing balance is shown in Figure 1. The number of patients able to maintain standing balance was lower with increasing difficulty of the standing positions, both for eyes open and eyes closed conditions. In tandem stance with eyes closed 4 (2%) patients were able to maintain balance. Comparable percentages were found for the subgroup who underwent additional continuous blood pressure measurements as shown in Figure 1B. Table 1 shows that 45% of the patients reported impaired standing balance and 65% of the patients reported at least one fall incident in the 12 months prior to the visit to the outpatient clinic.

### Blood pressure measures and standing balance

#### Intermittent blood pressure measurements

The associations between intermittent blood pressure measures and the ability to maintain standing balance adjusted for age and sex are presented in Table 2. In standing positions with eyes open, intermittent blood pressure measures were not associated with the ability to maintain balance. In standing positions with eyes closed, intermittent blood pressure measures, except OH_intermittent_, were not associated with the ability to maintain standing balance. Patients with OH_intermittent_ were significantly less likely to be able to maintain balance in semi-tandem stance with eyes closed. All intermittent blood pressure measures were not associated with self-reported impaired standing balance and history of falls as presented in Table S2. Additional adjustments for BMI, gait speed, MMSE score and handgrip strength did not influence the results.

**Table 2:** Association between intermittent blood pressure measures and the ability to maintain standing balance in all elderly patients (n = 197).

	Eyes open conditions						Eyes closed conditions					
	Side-by-side		Semi-tandem		Tandem		Side-by-side		Semi-tandem		Tandem	
	OR (95% CI)	p	OR (95% CI)	p	OR (95% CI)	p	OR (95% CI)	p	OR (95% CI)	p	OR (95% CI)	p
**Supine blood pressure** ^a^												
Systolic BP	1.01 (0.99–1.04)	.33	1.00 (0.98–1.01)	.65	1.00 (0.99–1.02)	.79	1.00 (0.98–1.01)	.91	1.00 (0.98–1.01)	.42		
Diastolic BP	1.04 (0.99–1.10)	.15	1.01 (0.97–1.04)	.76	1.00 (0.90–1.03)	.93	1.03 (0.99–1.07)	.13	1.01 (0.98–1.04)	.59		
**Blood pressure decrease after postural change**												
Orthostatic hypotension^b^	1.32 (0.25–7.01)	.75	1.10 (0.37–3.29)	.87	0.82 (0.31–2.17)	.69	0.66 (0.25–1.72)	.39	0.33 (0.12–0.89)	**.03**	n.a.	
*Systolic BP decrease ^c^*												
1 minute	1.04 (1.00–1.08)	.07	1.01 (0.98–1.03)	.51	1.01 (0.98–1.03)	.60	1.01 (0.99–1.03)	.48	1.00 (0.98–1.02)	.73		
3 minutes	1.02 (0.98–1.07)	.34	1.00 (0.98–1.03)	.86	1.01 (0.98–1.03)	.64	1.00 (0.98–1.03)	.84	0.99 (0.97–1.02)	.60		
*Diastolic BP decrease ^c^*												
1 minute	1.05 (0.96–1.14)	.32	1.05 (0.99–1.11)	.09	1.00 (0.96–1.05)	.88	1.03 (0.98–1.08)	.32	0.99 (0.95–1.04)	.75		
3 minutes	1.02 (0.93–1.12)	.63	1.01 (0.96–1.07)	.63	1.01 (0.96–1.05)	.82	0.99 (0.94–1.04)	.57	0.99 (0.95–1.03)	.63		

All data are from logistic regression analysis with adjustments for age and sex. Ability to maintain standing balance: 0  =  unable, 1  =  able. ^a^ Measured after at least 5 minutes in supine position. ^b^ Orthostatic hypotension: 0  =  absent, 1  =  present; defined as decrease in systolic blood pressure of ≥ 20 mmHg or decrease in diastolic blood pressure of ≥ 10 mmHg during 3 minutes after postural change. ^c^ Supine blood pressure minus blood pressure at 1 or 3 minutes after postural change. n.a.  =  not applicable, number of elderly patients able to maintain this balance condition is less than 5.

#### Continuous blood pressure measurements

**Figure 2:**
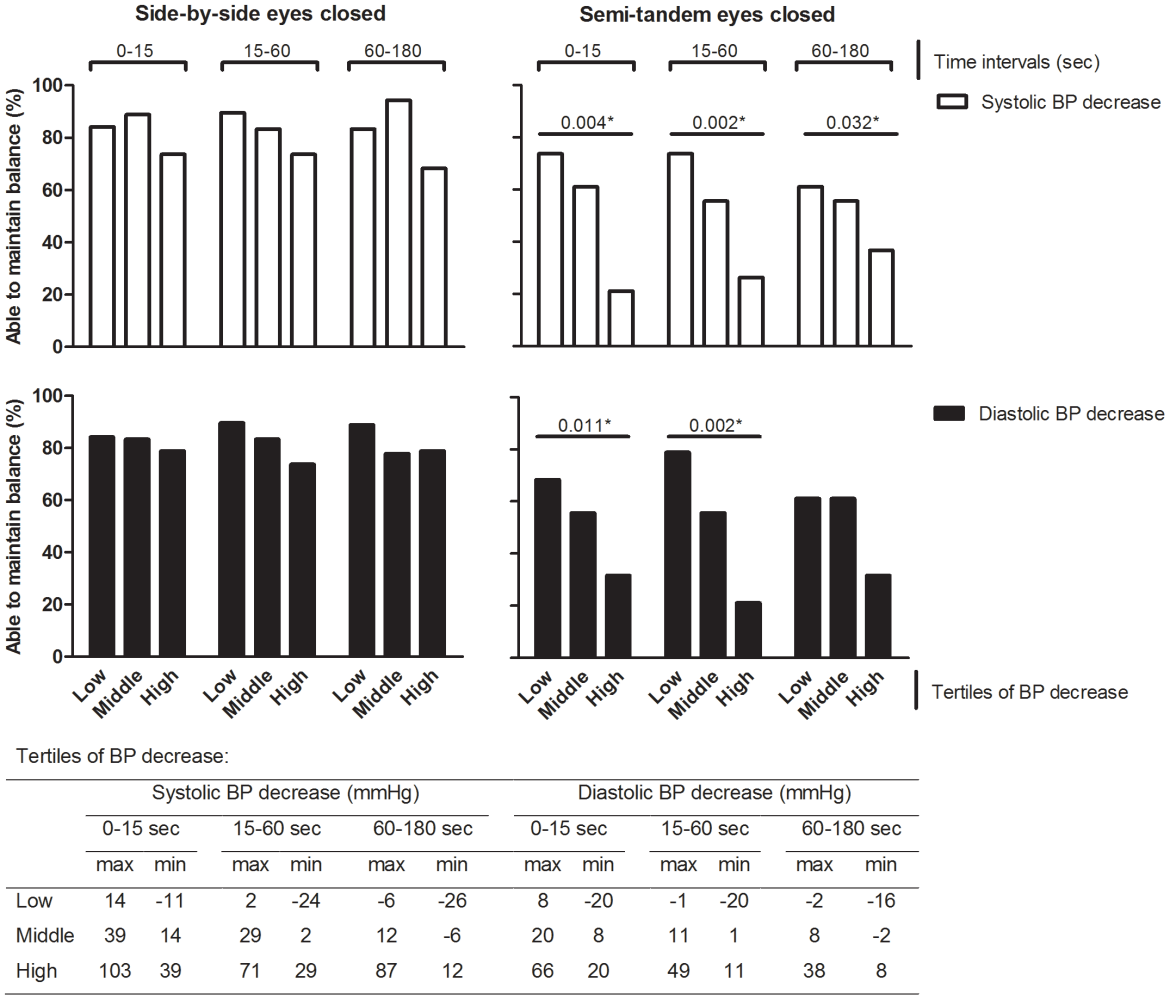
Percentage of elderly patients able to maintain balance during side-by-side and semi-tandem stance with eyes closed. Data is given for tertiles of systolic and diastolic blood pressure (BP) decrease, continuously measured, during the time period in seconds after postural change. *P values derived from logistic regression analyses with adjustments for age and sex.

The associations between continuous blood pressure measures and the ability to maintain standing balance adjusted for age and sex are displayed in Table S3. The main findings are visualized in Figure 2. In standing positions with eyes open, blood pressure measures were not associated with the ability to maintain balance. In standing positions with eyes closed, patients with a higher decrease in systolic blood pressure in each time period after postural change and patients with a higher decrease in diastolic blood pressure during the first 15 seconds or during 15 to 60 seconds after postural change were significantly less likely to be able to maintain balance in semi-tandem stance with eyes closed. Patients with OH_continuous_ were significantly less likely to be able to maintain balance in semi-tandem stance with eyes closed. Additional adjustments for BMI, gait speed, MMSE score and handgrip strength did not influence the results.

**Figure 3:**
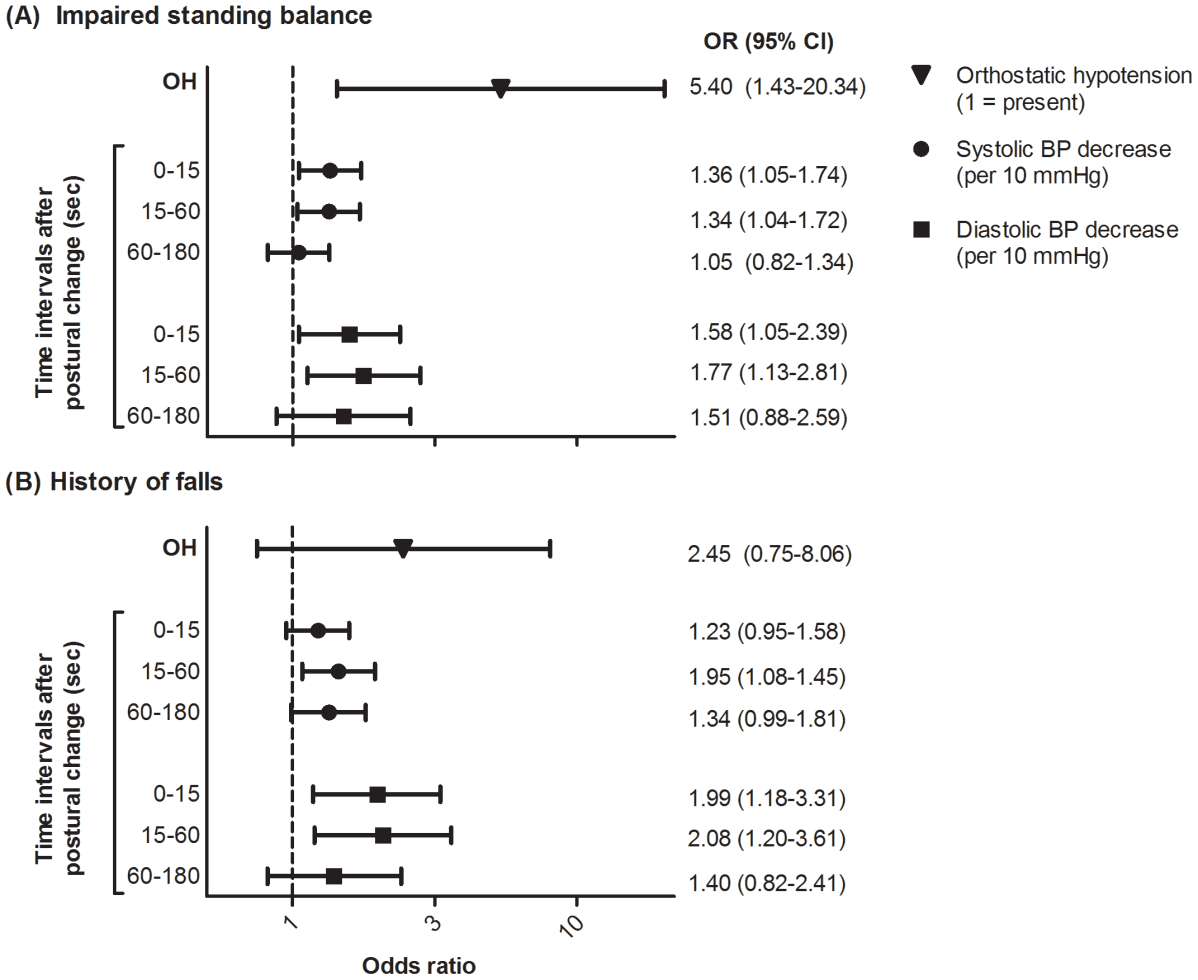
Forest plots of the association between blood pressure and A) reported impaired standing balance and B) history of falls. Blood pressure measures were determined with continuous measurements in subgroup who underwent additional continuous blood pressure measurements (n = 58). Orthostatic hypotension: 0  =  absent, 1  =  present; defined as a decrease in systolic blood pressure of ≥ 40 mmHg or in diastolic blood pressure of ≥ 20 mmHg during 15 seconds after postural change or a decrease in systolic blood pressure of ≥ 20 mmHg or diastolic blood pressure of ≥ 10 mmHg between 15 and 180 seconds after postural change. Reported impaired balance: 0  =  never or sometimes, 1  =  regularly or always. History of falls: 0  =  no falls, 1  =  falls. Results are presented in odds ratios per 10 mmHg blood pressure decrease and 95% confidence intervals with adjustments for age and sex. No overlap with 1.0 indicates a significant difference.

The associations between continuous blood pressure measures and self-reported impaired standing balance and history of falls adjusted for age and sex are displayed in Figure 3 using a forest plot. This plot shows the odds ratio and 95% confidence interval per association, in which no overlap with 1.0 indicates a significant difference. Patients with a higher decrease in systolic or diastolic blood pressure during the first 15 seconds or during 15 to 60 seconds after postural change were significantly more likely to report impaired standing balance. Patients with a higher decrease in systolic or diastolic blood pressure during 15 to 60 seconds after postural change were significantly more likely to experience falls in the last 12 months. In addition, patients with a higher decrease in diastolic blood pressure in the first 15 seconds after postural change were significantly more likely to have a history of falls. Patients with OH_continuous_ were significantly more likely to report impaired standing balance, but not to experience falls in the last 12 months. Additional adjustments for BMI, gait speed, MMSE score and handgrip strength did not influence the results.

## Discussion

Significant associations between continuously measured blood pressure decrease after postural change and the ability to maintain standing balance in conditions with eyes closed, self-reported impaired standing balance and history of falls were found in community-dwelling elderly referred to a geriatric outpatient clinic. Furthermore, OH determined with continuous measurements was associated with reduced ability to maintain standing balance and with increased self-reported impaired standing balance, but not with falls.

This is the first study that investigated the association of blood pressure measures with ability to maintain standing balance and self-reported impaired standing balance in elderly outpatients. In previous studies, no association was found between hypertension and quality of standing balance, measured by CoP movement, in healthy elderly [24]. However, hypertension has been associated with standing balance during a dynamic test, in which the patient was pulled backward and the response was quantified [25]. In this study, no association was found between blood pressure in supine position and measures of standing balance. Previous studies in healthy elderly and Parkinson patients found an association between OH, determined using blood pressure measurements at rest, after standing up and after one, two and three minutes of standing, and quality of standing balance, measured by CoM movement; elderly with OH were found to have an increased CoM movement during stance compared to elderly without OH [22], [23]. In accordance with those studies, we found an association of presence of OH and blood pressure decrease with subjective (i.e. self-reported impaired standing balance) and objective (i.e. ability to maintain standing balance) measures of standing balance.

Previous studies investigated the association between blood pressure measures and falls. In this study continuous blood pressure measures did associate with falls, which is conflicting with other studies [9], [12], [28]. In accordance with other studies, no association was found between intermittent blood pressure measures and falls [8], [11], [28]. Conflicting results could be due to variance in assessment and the lack of an uniform definition of OH. Furthermore, falls were assessed in different ways, i.e. retrospective, self-reported versus prospective assessment during a follow up period or use of self-administrated fall risk profiles.

The association between blood pressure decrease and reduced ability to maintain standing balance may be explained by cerebral hypoperfusion. Cerebral autoregulation modulates cerebral blood flow and cerebral perfusion in order to maintain sufficient oxygenation of the brain regions with fluctuations in blood pressure [34] and is affected by impaired blood pressure regulation [35], [36]. As a result, rapid or large decreases in blood pressure may lead to a decrease in cerebral blood flow [37]–[39], which increases the risk of repetitive transient hypoperfusion of the brain resulting in ischemic brain damage and impaired neural control [40]–[43]. As neural control is involved in standing balance, this can result in impaired standing balance. This hypothesis is supported by previous findings of a negative association between ischemic brain damage quantified by white matter hyperintensities on magnetic resonance imaging (MRI) and the ability to maintain balance during specific conditions [42], [44]–[46]. Furthermore, white matter hyperintensities were associated with higher CoP movement which is assumed to reflect poor quality of standing balance [47]. An alternative explanation may be a common-cause, i.e. impaired blood pressure regulation and impaired standing balance both are the result of the same factor, e.g. comorbidities, neurodegeneration or cerebrovascular lesions without a direct causal relation. Further research is needed to get better insight in the causal underlying mechanisms between blood pressure and standing balance.

The association between blood pressure decrease and the ability to maintain standing balance became apparent in standing positions with eyes closed. During this specific standing condition, the nervous system has to compensate for the elimination of visual information by use of sensory reweighting [48]. The sensory systems deteriorates with increasing age [49] and elderly have to rely on less accurate and reliable sensory information in case of elimination of the visual information, which makes standing with eyes closed more difficult. Besides the sensory systems involved in standing balance, sensory systems involved in blood pressure regulation, e.g. baroreceptors, deteriorate with age and age related diseases [50], [51]. This is a possible explanation for the fact that the association between blood pressure decrease and the ability to maintain standing balance was only present in standing positions with eyes closed.

The association between blood pressure decrease and standing balance was detected using objective (i.e. the ability to maintain standing balance) as well as subjective measures of standing balance (i.e. self-reported impaired standing balance and history of falls). Comparable results for the ability to maintain balance and falls were found, as impaired standing balance is a risk factor for falls[13], [19], [20]. Comparable results between the ability to maintain balance and self-reported impaired balance confirm the relation between the subjective and objective measures of standing balance and strengthen the clinical value of the outcome.

No association was observed between supine blood pressure and the ability to maintain standing balance, self-reported impaired standing balance or history of falls. However, previous research showed that hypertension, measured in sitting position, was associated with an increase in brain damage and concurrent impairments in mobility, cognition and mood in elderly with a mean age of 75 years [40], [42]. These conflicting results might be explained by age differences. In the very old (aged above 85 years) high blood pressure is associated with better survival, mediated by poor health status and frailty in the subject with lower blood pressure. In contrast, high blood pressure in a younger population (mean age 74 years) is associated with poor survival[18]. It is unknown if there is a certain age or state of cardiovascular disease in which a high blood pressure becomes of benefit due to better perfusion. A next step would be to focus on different age groups, which will be of clinical added value. This requires large study sample sizes.

In this study, the largest decrease in blood pressure was found during the first 60 seconds after postural change by use of continuous blood pressure measurements, which is in accordance with previous research [12]. Using intermittent blood pressure measurements only one time point is recorded, which has as consequence that peak blood pressure decreases may be missed. In this study, OH determined with intermittent measurements was present in 15 percent of the patients compared to 57 percent of the patients when OH was established with continuous measurements, which is in accordance with previous findings [52]. Seventy-nine percent of these elderly were established as OH patients only with continuous measurements. The use of intermittent measurements may therefore underestimate the number of OH patients.

Strength of this study was the unique study population of elderly patients. No exclusion criteria were applied. The population is representative for the community-dwelling elderly visiting the geriatric outpatient clinic. Furthermore, the use of continuous blood pressure measurements provided additional information about the blood pressure during the first 60 seconds after postural change and made it possible to include iOH in the analyses. As blood pressure was measured during 3 minutes after postural change, delayed OH, which occurs ten minutes or more after postural change [53], could not be measured. Limitation of this study is the cross-sectional design, which makes it impossible to draw conclusions about a causal relation between blood pressure regulation and standing balance. Furthermore, history of falls was measured using questionnaires which could result in recall bias. Despite the lower number of patients with continuous blood pressure measurements, we were able to find valuable associations of blood pressure decrease with standing balance.

## Conclusions

In conclusion, only by using continuous blood pressure measurements as a proxy for blood pressure regulation, associations with the ability to maintain standing balance, self-reported impaired standing balance and history of falls were found. The fact that previous associations could not be detected with intermittent blood pressure measurements, demonstrates the additional value of continuous over intermittent blood pressure measurements in routine geriatric assessment.

## Supporting Information

Table S1 **Blood pressure measures determined with continuous measurements in subgroup of elderly patients who underwent additional continuous blood pressure measurements (n = 58).**
(DOC)

Table S2 **Association between blood pressure measures determined with intermittent measurements and reported impaired standing balance and history of falls in all elderly patients (n = 197).**
(DOC)

Table S3 **Association between blood pressure measures determined with continuous measurements and the ability to maintain standing balance in subgroup of elderly patients who underwent additionally continuous blood pressure measurements (n = 58).**
(DOC)

Database S1 **Database of 197 elderly referred to a geriatric outpatient clinic consisting of blood pressure data measured intermittently and continuously and standing balance data.**
(SAV)
