# Supplementary material for: Blood Pressure Associates with Standing Balance in Elderly Outpatients
Source: PLoS One. 2014 Sep 15;9(9):e106808. doi: 10.1371/journal.pone.0106808 (PMC4164445; doi:10.1371/journal.pone.0106808)
Supplement: Table S1 — Blood pressure measures determined with continuous measurements in subgroup of elderly patients who underwent additional continuous blood pressure measurements (n = 58). (DOC) [file pone.0106808.s001.doc]

Table S1. Blood pressure measures determined with continuous measurements in subgroup of elderly patients who underwent additional continuous blood pressure measurements (n = 58).

|  | Subgroup (n = 58) |
| --- | --- |
| **Supine blood pressure** ^a^ |  |
| Systolic blood pressure, mmHg | 152 (27) |
| Diastolic blood pressure, mmHg | 75.9 (14.5) |
| **Blood pressure decrease after postural change** |  |
| Orthostatic hypotension, continuously measured; n (%) ^b^ | 33 (56.9) |
| *Systolic blood pressure decrease, mmHg ^c^* |  |
| 0 to 15 seconds | 29.0 (25.0) |
| 15 to 60 seconds | 17.4 (24.1) |
| 60 to 180 seconds | 8.4 (23.2) |
| *Diastolic blood pressure decrease, mmHg ^c^* |  |
| 0 to 15 seconds | 15.0 (15.5) |
| 15 to 60 seconds | 7.48 (14.35) |
| 60 to 180 seconds | 4.40 (11.82) |

All variables are presented as mean with standard deviation unless indicated otherwise. ^a^ Mean blood pressure in supine position of the last 60 seconds before postural change. ^b^ Orthostatic hypotension defined as decrease in systolic blood pressure of ≥ 40 mmHg or diastolic blood pressure of ≥ 20 mmHg during 15 seconds after postural change or decrease in systolic blood pressure of ≥ 20 mmHg or diastolic blood pressure of ≥ 10 mmHg between 15 and 180 seconds after postural change. ^c^ Supine blood pressure minus lowest blood pressure measured in the time period after postural change.
