# Supplementary material for: Blood Pressure Associates with Standing Balance in Elderly Outpatients
Source: PLoS One. 2014 Sep 15;9(9):e106808. doi: 10.1371/journal.pone.0106808 (PMC4164445; doi:10.1371/journal.pone.0106808)
Supplement: Table S2 — Association between blood pressure measures determined with intermittent measurements and reported impaired standing balance and history of falls in all elderly patients (n = 197). (DOC) [file pone.0106808.s002.doc]

Table S2. Association between blood pressure measures determined with intermittent measurements and reported impaired standing balance and history of falls in all elderly patients (n = 197).

|  | Reported impaired balance | | |  | History of falls | | |
| --- | --- | --- | --- | --- | --- | --- | --- |
|  | OR (95% CI) | | p |  | OR (95% CI) | | p |
| **Supine blood pressure ^a^** |  |  |  |  |  |  |  |
| Systolic BP | 1.01 (1.00-1.03) | | .05 |  | 1.01 (1.00-1.03) | | .04 |
| Diastolic BP | 1.01 (0.98-1.04) | | .51 |  | 1.00 (0.97-1.03) | | .82 |
| **Blood pressure decrease after postural change** | | | | | |  |  |
| Orthostatic hypotension ^b^ | 2.17 (0.95-4.95) | | .06 |  | 1.70 (0.70-4.16) | | .24 |
| *Systolic BP decrease ^c^* |  |  |  |  |  |  |  |
| 1 minute | 1.02 (1.00-1.03) | | .13 |  | 1.01 (0.99-1.03) | | .20 |
| 3 minutes | 1.01 (0.99-1.04) | | .16 |  | 1.01 (0.99-1.03) | | .29 |
| *Diastolic BP decrease ^c^* |  |  |  |  |  |  |  |
| 1 minute | 1.02 (0.98-1.07) | | .31 |  | 1.02 (0.97-1.06) | | .47 |
| 3 minutes | 1.01 (0.97-1.05) | | .63 |  | 1.01 (0.97-1.05) | | .71 |

All data are from binary logistic regression analysis with adjustments for age and sex. Reported impaired standing balance: 0 = never or sometimes, 1 = regularly or always. History of falls: 0 = no falls, 1 = falls. ^a^ Measured after at least 5 minutes in supine position. ^b^ Orthostatic hypotension 0 = absent, 1 = present defined as a decrease in systolic blood pressure of ≥ 20 mmHg or diastolic blood pressure of ≥ 10 mmHg during 3 minutes after postural change. ^c^ Supine blood pressure minus blood pressure at 1 or 3 minutes after postural change.
