# Supplementary material for: Blood Pressure Associates with Standing Balance in Elderly Outpatients
Source: PLoS One. 2014 Sep 15;9(9):e106808. doi: 10.1371/journal.pone.0106808 (PMC4164445; doi:10.1371/journal.pone.0106808)
Supplement: Table S3 — Association between blood pressure measures determined with continuous measurements and the ability to maintain standing balance in subgroup of elderly patients who underwent additionally continuous blood pressure measurements (n = 58). (DOC) [file pone.0106808.s003.doc]

Table S3. Association between blood pressure measures determined with continuous measurements and the ability to maintain standing balance in subgroup of elderly patients who underwent additionally continuous blood pressure measurements (n = 58).

|  | Eyes open conditions | | | | | | | |  | Eyes closed conditions | | | | | | | | | |
| --- | --- | --- | --- | --- | --- | --- | --- | --- | --- | --- | --- | --- | --- | --- | --- | --- | --- | --- | --- |
|  | Side-by-side | |  | Semi-tandem | |  | Tandem | |  | Side-by-side | | |  | Semi-tandem | | |  | Tandem | |
|  | OR (95% CI) | p |  | OR (95% CI) | p |  | OR (95% CI) | p |  | OR (95% CI) | | p |  | OR (95% CI) | | p |  | OR (95% CI) | p |
| **Supine blood pressure** ^a^ | | | | | | | | |  |  |  |  |  |  |  |  |  |  |  |
| Systolic BP | 0.99 (0.94-1.04) | .69 |  | 0.99 (0.97-1.02) | .67 |  | 0.99 (0.96-1.01) | .24 |  | 0.97 (0.95-1.00) | | .07 |  | 0.99 (0.97-1.01) | | .28 |  |  |  |
| Diastolic BP | 1.07 (0.95-1.21) | .28 |  | 1.05 (0.98-1.11) | .15 |  | 1.00 (0.96-1.05) | .94 |  | 0.97 (0.95-1.00) | | .07 |  | 0.99 (0.97-1.01) | | .28 |  |  |  |
| **Blood pressure decrease after postural change** | | | | | | | | |  |  |  |  |  |  |  |  |  |  |  |
| Orthostatic hypotension ^b^ | 6.30 (0.29-135) | .24 |  | 1.40 (0.30-6.62) | .67 |  | 0.46 (0.13-1.57) | .22 |  | 0.52 (0.11-2.41) | | .40 |  | 0.18 (0.05-0.62) | | **.007** |  |  |  |
| *Systolic BP decrease ^c^* | | | | | | | | |  |  |  |  |  |  |  |  |  | n.a. |  |
| 0 to 15 sec | 1.03 (0.96-1.11) | .36 |  | 1.00 (0.97-1.03) | .80 |  | 0.99 (0.96-1.01) | .22 |  | 0.98 (0.95-1.00) | | .10 |  | 0.96 (0.94-0.99) | | **.004** |  |  |  |
| 15 to 60 sec | 1.02 (0.95-1.08) | .65 |  | 0.99 (0.96-1.02) | .38 |  | 0.98 (0.95-1.01) | .13 |  | 0.97 (0.95-1.00) | | .08 |  | 0.96 (0.93-0.98) | | **.002** |  |  |  |
| 60 to 180 sec | 0.99 (0.93-1.06) | .83 |  | 1.02 (0.98-1.06) | .45 |  | 0.98 (0.94-1.01) | .16 |  | 0.97 (0.95-1.00) | | .09 |  | 0.97 (0.94-1.00) | | **.03** |  |  |  |
| *Diastolic BP decrease ^c^* | | | | | | | | |  |  |  |  |  |  |  |  |  |  |  |
| 0 to 15 sec | 1.12 (0.99-1.27) | .08 |  | 1.03 (0.98-1.09) | .30 |  | 1.00 (0.96-1.04) | .83 |  | 0.97 (0.93-1.02) | | .25 |  | 0.95 (0.91-0.99) | | **.01** |  |  |  |
| 15 to 60 sec | 1.13 (0.98-1.31) | .10 |  | 1.02 (0.96-1.08) | .49 |  | 0.97 (0.93-1.02) | .24 |  | 0.96 (0.91-1.01) | | .08 |  | 0.91 (0.86-0.97) | | **.002** |  |  |  |
| 60 to 180 sec | 1.02 (0.89-1.17) | .77 |  | 1.09 (0.99-1.19) | .07 |  | 0.97 (0.92-1.03) | .34 |  | 0.96 (0.91-1.02) | | .23 |  | 0.96 (0.90-1.01) | | .09 |  |  |  |

All data are from logistic regression analysis with adjustments for age and sex. Ability to maintain balance: 0 = unable, 1 = able. ^a^ Mean blood pressure in supine position of the last 60 seconds before postural change. ^b^ Orthostatic hypotension: 0 = absent, 1 = present; defined as a decrease in systolic blood pressure of ≥ 40 mmHg or in diastolic blood pressure of ≥ 20 mmHg during 15 seconds after postural change or a decrease in systolic blood pressure of ≥ 20 mmHg or diastolic blood pressure of ≥ 10 mmHg between 15 and 180 seconds after postural change. ^c^ Supine blood pressure minus lowest blood pressure measured in the time period after postural change. n.a. = not applicable, number of elderly patients able to maintain this balance condition is less than 5.
